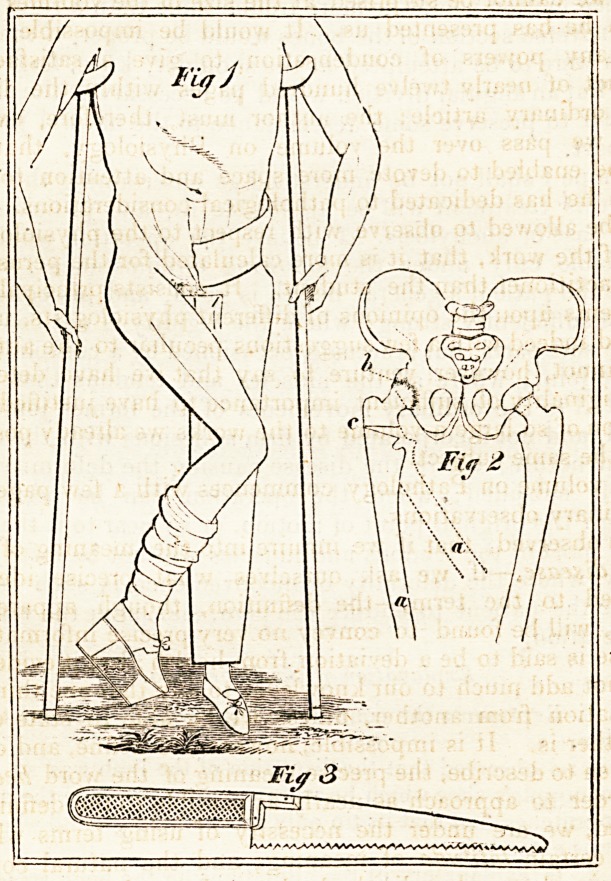# On the Treatment of Anchylosis, by the Formation of Artificial Joints

**Published:** 1827-08

**Authors:** J. Rhea Barton

**Affiliations:** Surgeon to the Pennsylvania Hospital.


### ANCHYLOSIS.

*On the Treatment of Anchylosis, by the Formation of Artificial Joints.* By J. Rhea Barton, M.D. Surgeon to the Pennsylvania Hospital. (Condensed from the *North American Medical and Surgical Journal.*)

[WITH A WOOD-CUT.]

I BEG leave to call the attention of my professional brethren to the following paper, believing that it contains some new views, in relation to a deformity and lameness, hitherto, I think, excluded from the surgeon's list of curable complaints, and one of the opprobria of our art: I allude to a firm, bony anchylosis of the joints.

It is well known that no such deformity can be established until the original natural structure of a joint shall have undergone an entire change. The cartilages must previously be absorbed, leaving only the two rough ends of the bones to unite and become incorporated, and as it were one bone. It is not surprising, therefore, that we should not have bestowed upon such defects a second thought, in reference to a cure; since parts, once gone, cannot in living matter be replaced, as they may be in machinery of human construction. The restoration, therefore, of a natural joint, once destroyed, being impossible, what has always been our course to persons thus afflicted? It has been to apprise them of their irreparable loss, leaving time only to reconcile them to a misfortune entailed on them through after-life. Having witnessed, about sixteen months ago, a most distressing instance of deformity and lameness, from an injury of the hip-joint, it aroused me to much reflection on this subject, which ended in my adopting views, and a course of practice, which shall hereafter be detailed. I will relate the case referred to.

John Coyle, native of Philadelphia, twenty-one years of age, sailor on board the schooner Topaz, Captain Schyler, states that, on the 17th day of March, 1825, he fell from the hatchway into the ship's hold, upon the end of a barrel, a distance of about six or seven feet; that the force of the fall was sustained on the outside of his right hip; violent pain was the immediate consequence, and much tumefaction ensued; that after the injury he arose with difficulty, and attempted to walk; thinks he made one or two steps, but was compelled to retire to his hammock, where he lay contracted for the space of about eighteen days; was then taken into Porto Cavello, and conveyed to the hospital. When lodged upon his bed, he placed himself on his side, with the injured limb uppermost, drawing the thigh to a right angle with the axis of the pelvis, and the knee resting on the sound side. In this posture he continued, without any material alteration, for the space of about five months; in the meantime enduring all the suffering attendant upon a high degree of inflammation of one of the largest joints in the human body, and unalleviated by the support of splints, or a judicious antiphlogistic course of treatment. As might naturally be expected, a rigid and deformed limb was the result of such disease, combated only by the administration of some simple liniment.

With regard to the real nature of the primary injury sustained, little can be said. The opinion on this point of the medical attendant, under whose special care the patient was placed in the hospital, is not known. Dr. Murphy, surgeon general, who occasionally saw him, believed it to be a dislocation. On board of the Topaz, previous to his removal to the hospital, two physicians, belonging respectively to an English and French vessel of war, laying in port, inspected the limb, in company with the American consul Dr. Litchfield. Two of these gentleman thought there was fracture; the French physician believed it to be some form of luxation. It is certain, therefore, from the difference of sentiment, that there was much obscurity in the case.

In October, 1825, Coyle returned to Philadelphia, having been sent home by our consul.

Early on his arrival, he exhibited himself to me. He was then supported by crutches, having the thigh drawn up nearly to a right angle with the axis of the pelvis, and the knee turned inward, and projecting over the sound thigh; so that the outside of the foot presented forward. There was considerable enlargement round the hip, which so much obscured the case, even at this date, as to prevent me from forming any positive opinion as to the real nature of the original injury. From the fixed and immovable condition of the limb, it was impossible to ascertain whether, in a straight position, there would be shortening, and, if any, to what extent. The general feature of the limb bore somewhat the resemblance of that resulting from a dislocation into the ischiatic notch; yet the position in which the great trochanter stood, in relation to the superior anterior spinous process, discouraged such a belief. All things considered, I was rather inclined to the opinion that there had been neither fracture nor luxation, but that the violence of the fall had produced an extensive contusion of the round ligament and joint, and that disorganisation had followed the consequent inflammation. On this point, whatever might have been the nature of the accident, I thought I might feel assured that now all articular movement was gone, and that true anchylosis had taken place. Trusting, however, to the fallibility of my judgment, and wishing, for the patient's sake, that it might prove erroneous, I was induced to admit him into the Pennsylvania hospital, with the view of employing extension of the limb for some weeks, in hopes that its malposition might thereby be corrected. A perseverance, however, in this treatment, only proved the unalterable state of the hip-joint, and confirmed my early-formed opinion. He subsequently fell under the care of my colleagues, Drs. Hewson and Parrish, in their respective tours of surgical attendance in the hospital, where we several times considered his case in consultation, and were united in our final decision that any further attempts to release the joint would be useless.

Finding Coyle still in the hospital a year after his admission, reflection on his case led me to propose to my colleagues the following operation,—viz. To make an incision through the integuments, of six or seven inches in length, one half extending above and the other below the great trochanter; this to be met by a transverse section, of four or five inches in extent; the two forming a crucial incision, the four angles of which were to meet opposite to the most prominent point of the great trochanter; then to detach the fascia, and, by turning the blade of the scalpel sideways, to separate anteriorly all muscular structure from the bone, without unnecessarily dividing their fibres. Having done this, in like manner, behind and between the two trochanters, to divide the bone transversely through the great trochanter, and part of the neck of the bone, by means of a strong and narrow saw made for the purpose; this being accomplished, to extend the limb, and dress the wound. After the irritation from the operation shall have passed away, to prevent, if possible, by gentle and daily movement of the limb, &c. the formation of bony union; and to establish an attachment by ligament only, as in cases of ununited fractures, or artificial joints, as they are called.

In this proposition four material points presented themselves for consideration: viz.—the practicability of the operation; the degree of risk to life consequent thereto; the probability of being able to arrest ossific reunion; and the reasonable prospect of benefiting the patient thereby. The arguments I adduced in favour of such an operation were these:—That the anatomy of the part did not present any insurmountable obstacle to it. The fear of cutting into a joint was not to be entertained here, since, from previous disease, all the characteristics of a joint were gone; synovial membrane destroyed, cartilages absorbed, and an amalgamation of the head of the femur with the acetabulum had taken place. That the shock to the vital system would not, probably, be greater than is frequently endured from accidental injuries and other operations. That, if the opinion commonly assigned as the cause of the formation of false joints after fractures be true, such as frequent motion in the broken ends of the bone, a deficiency of tone in the system, &c. these agents could be resorted to with promising results.

In order to decide the important question as to the benefit which the patient might reasonably be expected to derive from such an operation, it was necessary to consider how nearly a joint, thus artificially formed, would resemble, in its construction and functions, the natural articulation. What change the divided ends of the bone would undergo; whence would be derived its cartilaginous surfaces, its ligaments, its capsule, and its synovia; and, finally, what was to restrain its undue motions. My hopes of improving his condition were founded upon the following facts and observations in relation to these points:—That a bone once divided, in a person otherwise healthy, must again unite, either by bone or by ligament; no case, to my knowledge, being on record where a broken bone remained always afterward destitute of attachment between the divided extremities, except in cases where one of the fragments has been so small, or so scantily supplied with blood, as to be unable to contribute its part in the restorative process; being sufficiently vascular only to retain its own vitality, as in case of the separation of the head of a bone. If, therefore, ossific union should be arrested, ligamentous adhesions would maintain the connexion. Writers observe, and it is confirmed by my own experience, that, when a fracture does not become consolidated, in the course of time the rugged edges are removed by absorption; the separated ends become condensed, smooth, and polished, and tipped with a kind of cartilaginous substance; they are likewise enclosed within a sort of capsule. Observation has also proved to me that this ligamentous structure, formed around and connecting the ends of an old fracture, is possessed of great strength; so much so, that I have, in several instances, witnessed persons sustaining the entire weight of their bodies on the ligaments of a false joint, requiring only lateral support to the limb. The freedom and latitude of motion in such cases, and total insensibility to pain, after a sufficient lapse of time, I had also witnessed, and were encouraging arguments. In the operation here proposed, no such great strength of ligaments as will support the body would be required; since, from the transverse section of the trochanter, bone will rest against bone, and strength in them sufficient only to prevent dislocation would be necessary. From my inquiries into the manner in which this joint was to be lubricated, I did not expect that a synovial membrane and fluid, in all their characters, would be generated; but ample proofs were not wanting of the immediate resources of nature in defending parts from injurious friction, in whatever part of the body it might be required, either by an exhalation from the adjacent structure, or by the intervention of a bursa. In the common false joint, where motion is discouraged as much as possible, sufficient moisture is there exuded to prevent painful attrition. It might reasonably be expected, therefore, that, where motion was continual, the lubricating moisture would be more abundantly exhaled. In ununited fractures, the false joint is uncontrolable, because there are no muscles specially adapted for its restraint; but, in the joint thus to be formed, the will alone must influence its movements, since nearly all the muscles which exercised their control over the original joint would be carefully preserved, to have a similar power over this; which is, in fact, a mere transfer of the point of articulation and resistance, from the head of the bone in the acetabulum to the upper end of the shaft of the femur, against the great trochanter.

Although I did not think it essential to the melioration of my patient's condition that the ends of the bone should at its section undergo any change, further than by the absorption of the asperities, I did believe that nature would not passively witness my labours to effect what she has so often herself endeavoured, unaided by art, to accomplish, but that she would be ready to co-operate with me, and to extend to completion that which human art alone would be incapable of,—the formation of a new and useful joint, as a substitute for that which disease had annihilated, either by the conversion of the trochanter into a socket, or by some more wise design. Dissections of old luxations and of fractures, near joints, present many ingenious and wonderful alterations of original, and depositions of new structure, to restore the functions and uses of parts impaired by accidents and disease. All authors notice these attempts at restoration. Sir Astley Cooper, in his Treatise on Dislocations and Fractures of the Joints, has particularly mentioned them, and given many interesting plates, illustrative of nature's unassisted achievements. Such circumstances strongly encouraged me in the experiment, and were considered as auguries of a favourable result.

These views were fully explained to my colleagues, and were accompanied by the assurance that my patient had been fairly apprised of his present condition, and of the nature and intentions of the operation proposed; that he had not merely acceded to it, but that, after placing his sufferings, the difficulties, risks to life, the chances of failure, and the dangers eventually of aggravated lameness, in the strongest and most exaggerated light, he had expressed his willingness to endure any pain or duration of suffering, and to subject himself to all hazards, for the remotest prospect of relief.

Accordingly, on the 22d of November, 1826, assisted by Drs. Hewson and Parrish, I proceeded to the operation publicly in the Pennsylvania hospital.

To a large medical class, and many respectable physicians assembled, I again represented the nature of the case and of the operation, and the views and course of reasoning which induced me to adopt it; stating, likewise, that I wished it to be distinctly understood that a submission to my contemplated plans had not been urged upon my patient by any false or delusory promises, but that an explanation of his existing condition, and of the means proposed to be attempted for his relief, were fully made to him, in language adapted to his right comprehension of the matter, as well by my colleagues as by myself; and that he had authorised me thus publicly to state that he was prepared to assume all and the exclusive responsibility for the issue.

The integuments and fascia being divided and raised, the muscles in contact with the bone, around part of the great trochanters were carefully detached, and a passage thereby made just large enough to admit of the insinuation of my fore-fingers before and behind the bone; the tips of which now met around the lower part of the cervix of the femur, a little above its root. The saw (see engraving, fig. 3,) was readily applied, and without any difficulty a separation of the bone was effected. The thigh was now released, and I immediately turned out the knee, extended the leg, and placed the limbs side by side; by a comparison of which, in reference to length, the unsound member betrayed a shortening of about half an inch. This might have been caused partly by a distortion of the pelvis. Not one blood-vessel required to be secured. Union by the first intention was not attempted; the lips of the wound were only supported by adhesive plaster and slight dressings. The patient was put to bed, and Desault's splints were applied to support the limb.

The operation, though severe, was not of long duration, it being accomplished in the space of about seven minutes.

In the evening, the patient suffered great pain, and was much prostrated; his pulse feeble, stomach irritable, with great restlessness.

Opium grs. ij. were given.

November 23d, *(following morning.)*—Vomiting inordinate; had a bad night; pulse feeble and irritable; limb painful, particularly along the fore part of the thigh. No nourishment retained. Some hemorrhage from the wound.

Prescriptions, during the day, Opium and Soda-water. In the evening, Opium and Camphor; Neutral Mixture.—Sinapism over the epigastric region.

24th.—Irritability of the stomach much allayed; pulse still frequent and feeble. Examined the wound superficially: no union; lips of wound much swollen and everted; very painful.

Prescriptions, Opium and cordial nourishment.

25th.—Stomach improved. Much pain in the bowels, which was relieved by a laxative enema.

Opium and milk-punch given.

26th.—Pulse very frequent and weak; wound not unfavourable.

Poultice applied.

27th.—Pulse as yesterday; not quite so much pain; approaching suppuration.

28th.—Some pus secreted.

30th.—Pulse frequent, but less irritation in it; wound suppurating.

December 1st.—Whole surface of wound covered with healthy granulations.

7th.—Granulations vigorous and contracting; matter copiously secreted from the cavity of the wound, and from under the fascia over the rectus muscle. This discharge I ascribed to the forcible separation of the rectus muscle from its aponeurotic covering, to which I supposed it had adhered in consequence of previous inflammation, and during the state of quietude and contraction of the limb.

Prescriptions, Decoction of Bark, Cinnamon, and Serpentaria. Milk-punch and Opium continued.—Simple dressings to the wound; compress and many-tailed bandage to the thigh.

20th.—Patient's limb and general health have been regularly improving until this evening, when, from some indiscretion in diet, he was assailed by a most violent flatulent colic, which resisted all the ordinary and powerful remedies. At the suggestion of Dr. Parrish, I resorted to dry syringing; and to this I attribute his relief.

21st.—Somewhat exhausted by last night's indisposition, otherwise comfortable. Wound cicatrising; pus diminishing in quantity.

January 20th, 1827.—During the past month, no circumstance has occurred, as to the patient's general health or the appearance of the wound, which deserves particular notice, except that the sore regularly diminished in size, and his strength increased. It must now be particularly noticed, that, in addition to the treatment already mentioned, after the twentieth day from the operation the limb was cautiously moved in such directions as resembled the natural movement of the sound hip-joint; but, in doing this, I was careful never to use such violence, to continue it so long at a time, or to repeat it so often, as to occasion any permanent irritation. A sufficient time was always allowed for the patient to recover from the soreness of the last motions, before the limb was again disturbed. At first it was necessary to allow an interval of several days between the movements, in order to obtain a subsidence of the soreness.

In the course of a short time, however, the part became more insensible to pain from this disturbance, and the limb was more frequently moved. During the last three weeks, the patient was requested, at my daily visits to him, to exert his muscles, in slightly flexing, extending, and rotating his thigh. This he accomplished without difficulty, and after a little practice without pain. As an auxiliary step, he was occasionally propped upright in bed, so that his pelvis might be at an angle with his thigh.

21st.—It is just sixty days since the operation was performed. The wound having now entirely healed, and all appearances of inflammation gone, Coyle, with careful assistance, left his bed, and, aided by crutches, stood erect, both feet reaching the floor: he thinks he bore ten or twelve pounds weight on the weakened limb for a few minutes; made an attempt to advance the leg, and did so exclusively by muscular exertion; then rested on the sound side, and rotated the knee, and says without pain. He was then placed in a wheeled chair, and moved to the fire, where he sat, with both feet down, for about an hour; then retired to bed.

22d.—Last night the limb felt a little sore, from the exertion of the preceding day; otherwise well. Considerable fluctuation discoverable along the direction of the rectus femoris.

23d.—A quantity of synovial fluid escaped by a very small sinuous opening in the cicatrix, unaccompanied by pain or other inconvenience. This discharge evidently came from the theca of the rectus femoris, and appeared to have been secreted in such superabundance in consequence of the great excitement produced in this structure, after having been so long in a state of inaction. Patient in other respects doing well.

26th.—Coyle arose, and with the assistance of crutches made an attempt to walk. At this trial he fairly used the weakened limb, alternately with the sound one, four or five steps in progression, and was then seated in his chair. In the course of the day he repeated his attempt, and succeeded in walking backward and forward a room of ordinary size, using each limb alternately, and making fair and natural steps.

27th.—Patient, in the presence of the medical class of the hospital, walked around the room several times; then held out his crutches, showing that he was capable of sustaining much of the weight of his body on the limb, without pain. On being asked whether he felt as if he had at the hip solid support for his body, he answered in the affirmative.

28th.—Improved in walking, and other movements of the limb, and in general strength; was able to stand alone and firmly without crutches.

31st.—Patient dressed himself, and walked, with the assistance of his crutches, to the manager's apartment, a distance of about 150 feet. Dr. Hewson and myself now examined him particularly, to ascertain the muscular control he possessed over this newly-formed joint. We found him able to perform flexion and extension, abduction and adduction, rotation inward and outward, and able to cross the opposite foot: he had then, in fact, regained every movement which the limb originally possessed.

Feb. 8th.—Patient's strength, local and general, has been daily recruiting. To-day, he walked about ninety or one hundred yards; and with aid got into a gig, and rode to an extreme part of the city, a distance of about five miles; felt no pain, except at one or two unavoidable jolts; returned, and felt no fatigue.

12th.—Improvement regular; stands alone, and without support; can walk with two canes only.

13th.—During the night my patient, whilst dreaming, gave his limb a violent twist: in consequence of which, I found him this morning in excessive pain, accompanied by a good deal of headache and fever; the thigh bore marks of approaching inflammation.

In the course of the day, an erysipelatous blush, with tumefaction, was apparent in the vicinity of the joint, and on two or three insulated spots on the thigh.

The limb was kept perfectly quiet, and lead-water applied.

14th.—Fever much abated; erysipelas subsiding, but has caused the cicatrix slightly to ulcerate. No pain in the joint.

Emollient poultice applied.

15th.—Fever subsiding; erysipelas disappearing; no pain.

16th and 17th.—Doing well. Simple ointment substituted for poultice. Inflammation gone.

18th.—Patient left the bed, to resume his exercise; found himself much weakened by his recent attack, but no pain in the new joint. Motions continue unimpeded.

19th.—Patient up, and walking about; not yet recovered the strength which he had acquired previous to his erysipelatous attack.

24th.—Patient has been daily walking about, and gaining strength.

March 1st.—Since the last report, my patient has been rapidly gaining strength. His appetite is good. The ulcer occasioned by the erysipelas, which at no time was more than a mere abrasion of the surface, and not in the slightest manner affecting the joint, may be considered as well. He sleeps soundly, either on his back or side. He rises in the morning, and retires not until night; in the meantime amusing himself by exercise in walking, which he now begins to accomplish by the aid merely of a cane: time only seems to be required to enable him to walk without even this assistance. The following is the degree to which he can perform the movements of his limb with perfect ease: By measurement from a straight line, he can advance his foot twenty-four inches; in stepping backward, twenty-six inches; in abduction, twenty inches; in rotation inward, six inches; and outward, six inches.

*Anatomical changes in the limb.*—An entire destruction of the hip-joint; the head of the femur immovably fixed in its acetabulum by anchylosis; an artificial joint formed between the two trochanters and part of the neck of the bone; a quantity of dense ligament formed around and supporting this joint. The glutei medius and minimus, obturators externus and internus, and pyriformis, remain passive, and are of no further use, as their origin and insertion are at points between which there is no motion. The interior portion of the quadratus femoris was left attached below the section of the bone; but it is probable that its usefulness has likewise been destroyed by the alteration of structure, which these parts must have undergone in the formation of the new joint. The loss of these muscles is supplied by the action of those that originate above, and are inserted below, the artificial joint.

As the patient has regained every motion of the limb which he originally possessed, it is interesting to know what muscles have assumed the offices of those that have been lost. In my calculations previous to the operation, I took the following view of this subject.

Flexion and extension, I supposed, would be performed by all the muscles which had those actions on the former joint, except the iliacus internus and psoas magnus; the power of these as flexors I feared would be lost, from the mechanical disadvantage under which they would act, owing to their insertion so near to the part to be moved. Rotation outward would in future be performed by the action of the iliacus internus and psoas magnus; rotation inward, by the tensor vaginæ femoris; abduction, by the simultaneous action of the tensor vaginæ femoris and gluteus maximus; adduction, by the triceps adductor; and circumduction, by the alternate action of all the muscles of the thigh. This I believe to be the present state of muscular influence.

Upon examining for the space that usually exists between the projection of the great trochanter and the posterior margin of the acetabulum, it will be discovered that it has been filled up by an accumulation of osseous matter, apparently deposited there to prevent displacement of the bones, and to form something like a socket.

The outward contour of the hip resembles that of the sound side, except in the disfigurement occasioned by the extensive crucial incisions.

When the legs are extended, a slight shortening of the right limb is perceivable, but not sufficient to cause him to limp in walking. The thickness of the blade of the saw, taken from the length of the femur, and the subsequent change of the divided surfaces, might have caused this retraction of the limb. When the knees are drawn up, there is an increased shortening, which again disappears as they are extended. By placing the hand over the upper part of the trochanter, and moving the limb, that portion of the bone will be found perfectly at rest; but when the hand is lowered to the part where the joint has been formed, the articulation is satisfactorily felt. The sensation conveyed to the hand is not like that occasioned by two bare surfaces of bone rubbed against each other, but like that impaired by the motions in a natural and healthy joint.

Having now established the fact that an artificial joint can be substituted for the loss of the natural articulation at the hip, it becomes a matter of importance to ascertain how far the same principles are applicable to the formation of new joints in other parts of the body, where natural motion has been lost. My reflections on the point has not presented any forbidden circumstances; but it is not in every joint that the loss of motion would be sufficiently important to call for the aid of a painful operation. The most serious evil is sustained by the loss of the hip, knee, shoulder, elbow, great toe, and finger joints, and of the lower jaw; and these, I believe, may all come within the reach of amendment by an operation, if the muscles which move these respective joints are in a sound and efficient state. If they have been lost, it would be palpably wrong to form a joint, since its unrestrained motion would be more troublesome than a rigid limb. A transverse section of the bones would be proper, if the operation were to be attempted at the shoulder, knee, fingers, or toes; but an angular division would be necessary at the elbow, in order to preserve some resemblance to the natural joint at this part.

I hope I shall not be understood as entertaining the belief that this treatment will be applicable to, and judicious in, every case of anchylosis. I believe the operation would be justifiable only under the following circumstances: viz.—where the patient's general health is good, and his constitution is sufficiently strong; where the rigidity is not confined to the soft parts, but is actually occasioned by a consolidation of the joint; where all the muscles and tendons that were essential to the ordinary movements of the former joint are sound, and not incorporated by firm adhesions with the adjacent structure; where the disease causing the deformity has entirely subsided; where the operation can be performed through the original point of motion, or so near to it that the use of most of the tendons and muscles will not be lost; and, finally, where the deformity or inconvenience is such as will induce the patient to endure the pain, and incur the risks of an operation.

### *Explanation of the Engraving.*

Fig. 1. Represents the condition of the patient previous to the operation.

It will be observed that the distortion of the limb was so great that the cripple's shoe which he wore did not supersede the necessity for crutches, but that its tip only reached the ground when the ankle was extended.

Fig. 2. Is explanatory of the alterations in the bony structure, first by disease, and subsequently by the operation.

*a.* Two faint lines, representing the direction of the femur, in correspondence with the thigh, in fig. 1.

*b*. The head of the femur and acetabulum; all motion between them arrested by anchylosis.

*c*. The point at which the bone was transversely sawn through, and the triangular gap at the section, occasioned by bringing down the thigh.

*d*. The femur, restored to its natural position after the separation of the bone.

Fig. 3. The saw used for dividing the bone: the blade about six or seven inches in length, and thinner on the back than on the edge; the end smoothly rounded off, to avoid piercing parts before it; teeth widely set.

## Figures and Tables

**Fig 1 Fig 2 Fig 3 f1:**